# Novel methodology to measure pre-procedure antimicrobial prophylaxis: integrating text searches with structured data from the Veterans Health Administration’s electronic medical record

**DOI:** 10.1186/s12911-020-1031-5

**Published:** 2020-01-30

**Authors:** Hillary J. Mull, Kelly Stolzmann, Emily Kalver, Marlena H. Shin, Marin L. Schweizer, Archana Asundi, Payal Mehta, Maggie Stanislawski, Westyn Branch-Elliman

**Affiliations:** 1 VA Boston Healthcare System, Center for Healthcare Organization and Implementation Research (CHOIR), 150 S. Huntington Ave, Boston, MA 02130 USA; 2 Department of Surgery, Boston University School of Medicine, Boston, MA USA; 3 Center for Access and Delivery Research and Evaluation (CADRE), Iowa City VA Health Care System, Iowa City, Iowa USA; 4 University of Iowa Carver College of Medicine, Iowa City, Iowa USA; 5 Department of Medicine, Boston University School of Medicine, Boston, MA USA; 6 Boston Medical Center, Department of Medicine, Division of Infectious Diseases, Boston, MA USA; 7 VA Boston Healthcare System, Department of Medicine, Sections of Infectious Diseases and Cardiology, Boston, MA USA; 8 Seattle-Denver Center of Innovation for Veteran-Centered and Value-Driven Care, Seattle, Washington and Denver, Colorado USA; 9 Division of Biomedical Informatics and Personalized Medicine, University of Colorado School of Medicine, Aurora, Colorado USA; 10 Harvard Medical School, Boston, MA USA

**Keywords:** Antibiotic prophylaxis, Algorithm, Veterans health administration, Cardiac device procedure, Quality measurement

## Abstract

**Background:**

Antimicrobial prophylaxis is an evidence-proven strategy for reducing procedure-related infections; however, measuring this key quality metric typically requires manual review, due to the way antimicrobial prophylaxis is documented in the electronic medical record (EMR). Our objective was to electronically measure compliance with antimicrobial prophylaxis using both structured and unstructured data from the Veterans Health Administration (VA) EMR. We developed this methodology for cardiac device implantation procedures.

**Methods:**

With clinician input and review of clinical guidelines, we developed a list of antimicrobial names recommended for the prevention of cardiac device infection. We trained the algorithm using existing fiscal year (FY) 2008–15 data from the VA Clinical Assessment Reporting and Tracking-Electrophysiology (CART-EP), which contains manually determined information about antimicrobial prophylaxis. We merged CART-EP data with EMR data and programmed statistical software to flag an antimicrobial orders or drug fills from structured data fields in the EMR and hits on text string searches of antimicrobial names documented in clinician’s notes. We iteratively tested combinations of these data elements to optimize an algorithm to accurately classify antimicrobial use. The final algorithm was validated in a national cohort of VA cardiac device procedures from FY2016–2017. Discordant cases underwent expert manual review to identify reasons for algorithm misclassification.

**Results:**

The CART-EP dataset included 2102 procedures at 38 VA facilities with manually identified antimicrobial prophylaxis in 2056 cases (97.8%). The final algorithm combining structured EMR fields and text note search results correctly classified 2048 of the CART-EP cases (97.4%). In the validation sample, the algorithm measured compliance with antimicrobial prophylaxis in 16,606 of 18,903 cardiac device procedures (87.8%). Misclassification was due to EMR documentation issues, such as antimicrobial prophylaxis documented only in hand-written clinician notes in a format that cannot be electronically searched.

**Conclusions:**

We developed a methodology with high accuracy to measure guideline concordant use of antimicrobial prophylaxis before cardiac device procedures using data fields present in modern EMRs. This method can replace manual review in quality measurement in the VA and other healthcare systems with EMRs; further, this method could be adapted to measure compliance in other procedural areas where antimicrobial prophylaxis is recommended.

## Background

Post-surgical and post-procedural infections, including cardiac device infections following electrophysiology procedures, are highly morbid [1, 2]. These severe complications can be prevented with appropriate pre-incisional antimicrobial prophylaxis, which can reduce rates of infection by approximately 80% and are strongly recommended in multi-society guidelines [3–6]. Given the high morbidity of these infections and their preventability, pre-incisional antimicrobial compliance is an important quality metric used in most U.S. hospitals, including the Veterans Health Administration (VA). However, measurement of this key quality metric represents a significant challenge as, unlike most medications, antimicrobials administered in the operating room generally do not require that an order be placed. Thus, measurement systems that are effective for monitoring most medication orders cannot be applied to assess pre-incision prophylaxis. Previously, a time-consuming and expensive manual review process was undertaken to determine compliance with pre-incisional antimicrobial prophylaxis in select surgical procedures [7, 8]; however, there is currently no infrastructure for tracking compliance in non-surgical procedural areas (i.e., outside the operating room and by an interventionist as opposed to a surgeon) where prophylaxis is also recommended.

Identifying whether antimicrobial prophylaxis was administered has historically relied on manual chart review because an electronic method has several challenges. Some institutions designed their electronic medical record (EMR) to include structured data fields for ongoing antimicrobial stewardship [9, 10]; however this approach may be unfeasible in large healthcare systems with existing EMR systems and is often reliant on structured data about antimicrobial dispensing. Within most procedural and operating room settings, pre-incisional antimicrobial prophylaxis can be directly dispensed without documentation in the EMR of medication administration. Documentation of prophylaxis regimens may be present only in pre-procedural order sets without EMR documentation of administration or may only be present in clinician notes, such as the operative note or anesthesia records. This documentation style creates an opportunity for a novel clinical informatics approach combining structured (e.g., pre-procedural antimicrobial orders) and unstructured data (e.g., documentation of prophylaxis in clinical notes) to measure compliance with pre-incisional prophylaxis. Further, this method has the potential to support quality monitoring of non-surgical procedures, including cardiac device implantations, where little is known about compliance with guideline-concordant antimicrobial prophylaxis.

The goal of this study was to develop a methodology integrating structured and unstructured data elements found in modern EMRs to replace manual record review for quality measurement with an electronic tool to measure pre-incisional prophylaxis. We demonstrated the performance of this method using cardiac device implantation procedures in the VA. The VA, a national healthcare system with a robust EMR, is an ideal setting to conduct this work. In particular, VA’s Corporate Data Warehouse (CDW) includes extensive unstructured text data from clinical notes in the EMR, allowing us to access a large dataset to develop and validate our tool [11]. Methods combining structured and unstructured data for electronic measurement of pre-incision prophylaxis could be adapted for other settings, including for traditional surgical procedures.

## Methods

We established a method to accurately classify cardiovascular implantable electronic device (CIED) procedures with pre-incisional antimicrobial prophylaxis by 1) developing an algorithm in SAS and SQL software that combined structured and text data from the VA EMR, and 2) testing and validating the algorithm using manual chart review of fiscal year (FY) 2008–2015 cardiac device procedures from the VA Clinical Assessment Reporting and Tracking-Electrophysiology (CART-EP) program and from a national dataset reviewed by members of the study team. The VA Boston Institutional Review Board approved this research prior to data collection and analysis. The study was also approved by the VA CART-EP program.

### Data sources

The VA CART-EP program is a national quality program supporting all VA cardiac catheterization labs, where invasive cardiac procedures are performed. The program’s mission is to monitor and enhance the quality and safety of invasive cardiac procedures for Veterans through clinical analytics and information technology [12, 13]. Although reporting to the CART program is now mandatory for all invasive cardiac procedures, cardiac device procedures are voluntarily reported to the CART-EP application. During the study period, approximately 20% of cardiac device procedures performed across the national VA healthcare system were part of CART-EP. CART-EP does not collect information about pre- or post-procedural antibiotic use; however, these data were available as part of an earlier study that compared the effectiveness of different infection prevention strategies and used the gold-standard of manual review to extract data about pre-incision prophylaxis [14].

The data used in our algorithm were derived from the VA CDW, a single national data repository which is where structured data and clinical notes within the VA are stored [14, 15]. Data extracted from the VA CDW include antimicrobial orders and documentation of antimicrobial dispensing/administration as well as unstructured data, which include electronic clinical notes entered into the VA EMR. While notes have been available since FY2000, one VA facility does not push clinical note data from their EMR into the CDW. In addition, clinical notes that are not electronic (e.g., scanned documents in the EMR such as hand-written notes and PDF files of outside hospital records) are not available as searchable text data in CDW. We validated our algorithm using independent manual record review in the VA’s research EMR, VistAWeb [16].

### Potential sources of antimicrobial prophylaxis data

Within the VA EMR, four potential sources of antimicrobial prophylaxis documentation were identified. The first was in the computerized order entry system in the form of antimicrobial orders. These orders could be entered either in the outpatient setting prior to a planned procedure, or in the inpatient settings prior to an urgent or emergent procedure. The second was antimicrobial administration, as documented in the VA bar coding system for inpatient orders, and outpatient prescriptions orders in the outpatient setting. The third was in clinical notes entered as electronic text notes into the VA EMR; these could be any typed note signed within the system within the 7-day window prior to the procedure and including the procedure date. The fourth was clinical notes written on paper and scanned before being entered as an attachment into the EMR; these included hand-written anesthesia records. All of the first three data sources were included for potential inclusion in the electronic algorithm and all four sources were used to establish a gold standard for whether antimicrobial prophylaxis was appropriately administered.

### Study sample

There were 6497 cardiac device procedures, including implantations and revisions of permanent pacemakers, entered into the CART-EP application between FY2008–15. After the full cohort was created using the CART-EP program data, a sample of the procedures (*n* = 2102) underwent manual review; study team members extracted information about various infection prevention strategies, including type and administration of pre-operative antimicrobial prophylaxis. The comprehensive review strategy included: 1) review of clinical notes entered directly into the VA EMR, 2) review of orders and medication administration, and 3) review of scanned-in paper records, which include hand-written anesthesia and procedural notes. Details of the sampling procedure are previously described [14, 15]. Duplicate records and records without a verified procedure in the EMR corresponding to the date entered into CART-EP were excluded from the analysis.

### Algorithm development and refinement process

We developed the algorithm to detect previously identified cases with and without antimicrobial prophylaxis in our gold standard dataset over several stages. First, we combined an initial set of data elements available from the VA EMR. Then, we added more data elements, removed elements with weaker performance, and iteratively arrived at the combination of data elements that best identified whether antimicrobial prophylaxis was given. The final version became our electronic measurement algorithm. Specifically, the optimization process was as follows. We began by including all the available data elements, e.g., electronic clinician text note extraction only, orders only, administration only, then added timing of the searches (e.g., including or excluding the procedure date), and then considered the types of antimicrobials, including an evaluation of only intravenous antimicrobials versus inclusion of oral medications.

Three clinicians (AA, PM, WBE) developed a comprehensive list of antimicrobials potentially administered for pre-incisional prophylaxis prior to cardiac device placement. The list of antimicrobials was based on 1) recommendations from surgical prophylaxis guidelines, which includes suggestions for appropriate prophylaxis prior to cardiac device procedures, and 2) clinician review and expertise [17]. A list of antimicrobial names, including both generic and brand-names and variations to account for spelling errors, was then created and this list was used to search clinical notes for documentation of administration. (See Additional file 1 for a listing of the text search terms used.) The list was also mapped to structured data in the VA EMR and orders and administration of relevant medications were extracted. For structured variables, a 7-day window prior to the procedure date was used, based on common clinical practice patterns, which may include entry of the pre-procedural antimicrobial at a pre-procedural cardiology visit.

Algorithm performance was assessed as sensitivity (how many true positive cases were identified as positive by the algorithm) and specificity (how many true negative cases were identified as negative by the algorithm). Manually reviewed CART-EP data was used as the gold standard for algorithm development. To finalize the algorithm, all discordant cases (e.g., algorithm flagged positive/CART-EP manual review was negative or algorithm flagged negative/CART-EP manual review was positive) underwent a second round of manual review by a different reviewer (EK or WBE) to identify the reasons for the discordant flag and to classify the reasons for discordance through expert analysis and discussion among clinicians on the study team. We used these findings to adjust the algorithm to improve accuracy.

### Algorithm validation

We validated the algorithm by applying the relevant structured and unstructured data extracts to all cardiac device procedures performed in facilities with procedure volume ≥ 50 total cases within the VA healthcare system from FY 2016–17. A sample of these procedures underwent manual review (EK) to confirm whether the algorithm successfully identified whether antimicrobial prophylaxis was administered. Reasons for discordance were again assessed through expert assessment and discussion. We also conducted an analysis stratified by VA facility, to determine if facility-level effects were driving algorithm performance and accuracy.

## Results

### Algorithm development

The manually reviewed CART-EP dataset included 2102 procedures at 38 VA facilities with appropriately administered antimicrobial prophylaxis documented in 2056 cases (97.8%). We applied the various data elements described above (see Appendix for detailed descriptions of these elements) and found 1954 (93.0%) of the CART-EP cases had a positive text note search, 1899 (90.3%) had an antimicrobial order identified, and 150 (7.14%) had documentation of medication administration (Table 1). When text note searches were combined with antimicrobial orders, 2048 cases were flagged (97.4%); there were very few flags in the medication administration data (150, 7.2%) and adding this variable did not change either sensitivity or specificity as in all cases there was also an antimicrobial order present.

**Table 1:** Iterative performance of antimicrobial prophylaxis identification algorithm in development stage with gold standard CART-EP Program Data (manual review; *n* = 2102 procedures in 38 facilities)^a^

Data Elements in Algorithm	CART-EP-reviewed cardiac device procedures (*n* = 2102)	PPV (True flagged ‘yes abx’/All flagged ‘yes abx’)	NPV (True flagged ‘no abx’/All flagged ‘no abx’)	Sensitivity (All flagged ‘yes abx’/Total ‘yes abx’ *n* = 2056)	Specificity (All flagged ‘no abx’/Total ‘no abx’, *n* = 46)
Manual review	2056 (97.8%)	–	–	–	–
Text note searches	1954 (93.0%)	1930/1954 (98.8%)	22/148 (14.9%)	1930 (93.9%)	22 (47.8%)
Orders	1899 (90.3%)	1883/1889 (99.2%)	30/203 (14.8%)	1883 (91.6%)	30 (65.3%)
Administration	150 (7.14%)	150/150 (100%)	46/1952 (2.36%)	150 (7.30%)	46 (100%)
Text note searches + Orders	2048 (97.4%)	2019/2048 (98.6%)	17/54 (31.5%)	2019 (98.2%)	17 (37.0%)
Text note searches + Administration	1955 (93.0%)	1931/1955 (98.8%)	22/147 (15.0%)	1931 (93.9%)	22 (47.8%)
Orders + Administration	1901 (90.4%)	1885/1901 (91.7%)	30/201 (14.9%)	1885 (91.7%)	30 (65.2%)
Text note searches + Orders + Administration	2048 (97.4%)	2019/2048 (98.6%)	17/54 (31.5%)	2019 (98.2%)	17 (37.0%)
Round 2 Changes					
Text note searches - Exclude oral medications	1950 (92.8%)	1928/1950 (98.9%)	24/152 (15.8%)	1928 (93.8%)	24 (52.2%)
Limit list to common prophylaxis medications	2044 (97.2%)	2017/2044 (98.7%)	19/58 (32.8%)	2017 (98.1%)	19 (41.3%)
Exclude notes from the day of the procedure	823 (39.1%)	823/825 (99.8%)	44/1277 (2.09%)	823 (40.0%)	44 (95.7%)
Include term “prophylaxis” in text searches	2048 (97.4%)	2019/2048 (98.6%)	17/54 (31.5%)	2019 (98.2%)	17 (37.0%)

^a^CART-EP Program data included 2102 cardiac device procedures with manually collected data on antimicrobial prophylaxis; of these, 2056 cases (97.8%) received antimicrobials prior to incision. Shaded cells indicate the elements of the final algorithm; details of these data elements and what tables they were applied to in the VA EMR are available in Appendix

Some facilities routinely placed a drug order for the pre-operative antimicrobial during a pre-procedure visit into the VA computerized ordering system and then did not document type of antimicrobial in the clinician note about the procedure; however, there was documentation that “prophylaxis” or “antibiotics/antimicrobials” were administered prior to incision. Due to this documentation style, adjusting the algorithm to include orders entered into the computerized order entry system for an appropriate pre-operative antimicrobial substantially improved sensitivity, although specificity was slightly reduced (Table 1). Specificity of drug orders was limited primarily because antimicrobials could be prescribed for another reason (i.e., prescribed to treat an unrelated infection, such as bronchitis) and/or were administered after the procedure but none-the-less ordered during the pre-operative period.

We found 39 false negative and 27 false positive cases (66 procedures misclassified, 3.14%, Table 2). The most common reason for false-negatives was lack of documentation of a drug name and lack of an order in the clinical notes, but notation that pre-incisional prophylaxis was administered (e.g., procedure note stating, “antibiotics administered prior to incision” without specifying type, 27 cases; all of these were at the same facility). False negatives also included lack of documentation of a specific drug in the procedure and anesthesia records and order placed > 7 days prior to the procedure (4 cases). In these cases, the orders were placed weeks to months prior to the procedure and some cases may have been rescheduled during the intervening period, thus causing an unusually long lag time between the antimicrobial order and performance of the cardiac device procedure. Three cases were missed because the procedure note documenting administration of prophylaxis was entered after the procedure date; thus the algorithm measured the antimicrobial administration as post-procedural. One case was due to documentation of prophylaxis in paper records only, one case was due to incorrect procedure date entered into the VA EMR, and one case was due to incorrect procedure type entered (cardiac catheterization, not cardiac device procedure). We found no antimicrobial documentation in any notes in two cases, while in another case, no relevant clinical or pharmacy documentation was identified in the CDW.

**Table 2:** Reason for misclassification between antimicrobial prophylaxis algorithm and manual review^a^

Reasons	Algorithm Development (n procedures)		Algorithm Validation (n procedures)	
	False +	False -	False +	False -
Documentation drug was administered, but no name used in EMR or order		27		
Documentation of drug name in EMR and order but placed > 7 days pre-procedure		4		
Clinician note entered into CDW with date ≥1 day post-procedure		3		
Documentation of drug name composed on paper and scanned into CDW		1		39
Incorrect procedure date in CDW		1		1
Incorrect CPT code in CDW		1		
No documentation in EMR or CDW		2		
Antimicrobials administered post-procedure	21		2	
No documentation of drug type in EMR or CDW	2			
Patient on antimicrobials for unrelated reason	3			
Antimicrobials used as part of a flush or wash during the procedure, but not given systemically	5			
Total	27^b^	39	2	40

^a^Manually collected data on antimicrobial prophylaxis from 2102 procedures in the CART-EP dataset (used for algorithm development over two rounds), and 100 manually reviewed cases from the FY16–17 national sample of cardiac device procedures (used for algorithm validation)

^b^Some cases were false positives for multiple reasons

Among the 27 false positive flags, 21 had antimicrobials documented in the clinician procedure note but administered *after* the procedure (e.g., “give cefazolin x 3 doses post-procedure, then Keflex x 5 days” documented at the end of the note, with no pre-incision doses administered). Some cases were incorrectly flagged by the algorithm for several reasons, including documentation that prophylaxis was given but not which type (2 cases), patient on antimicrobials for unrelated reason (e.g., treatment of chronic obstructive pulmonary disorder or bronchitis) at the time of the procedure (3 cases), antimicrobials used as part of a flush or wash during the procedure, but not given systemically (5 cases), and documentation of antimicrobial allergy (e.g., cefazolin allergy) in the clinician procedure note, but no administration of the drug.

Based on the initial findings, several updates to the algorithm were made (Table 1, second stage). All duplicate entries were removed and cases with incorrect non-cardiac device procedures incorrectly coded were excluded. Then, several variations to optimize algorithm performance were tested. First, as many of the false-positive flags were due to oral antimicrobials administered post-procedure but documented and recommended in the clinician note about the procedure, we excluded antimicrobials rarely used for prophylaxis and agents that are only available in oral formulation from all data sources (text searches, orders, administration). Second, penicillin, which is rarely used for prophylaxis but commonly documented as an allergy, was also excluded from the text note searches. Third, antimicrobial orders were limited to those lasting < 24 h, to reduce the number of false-positives that arose from patients receiving antimicrobials for treatment of unrelated infections, which are generally prescribed for longer periods of time. Lastly, cases with antimicrobial orders placed on the day of the procedure but after the time of the procedure were excluded; post-procedure antimicrobial administration is both ineffective and against clinical guidelines and should not be used to assess whether a patient has received appropriate pre-procedural antimicrobial prophylaxis. The optimal algorithm, which included a limited set of antimicrobial orders and excluded post-incisional antimicrobials, had a sensitivity of 98.1% and a specificity of 41.3%.

### Algorithm validation

The final algorithm was applied to the validation cohort of VA patients who underwent cardiac device procedures from FY2016–17 (*n* = 18,903). Unlike procedures identified in CART-EP, which included 38 facilities, this cohort included all 65 VA facilities that performed at least 50 cardiac device procedures during the study period. The final algorithm identified pre-procedural antimicrobials in 16,606 cases (87.8%).

A random sample of 50 cases with a positive flag and 50 cases with a negative flag then underwent manual review; 48 positively flagged cases were true positives (sensitivity, 96%) whereas 10 of the negatively flagged cases were true negatives (specificity, 20%). The two false-positive cases were both due to documentation of antimicrobial administration in the clinical procedure notes but administered after the procedure (Table 2). The false negative cases (40) were primarily due to EMR documentation of antimicrobial administration only in scanned paper records that were not searchable by the algorithm (70%); this limitation was clustered by facility. Other false negative cases had mention of antimicrobial administration in the typed record, but the antibiotic name was only mentioned in scanned notes that were not available for electronic text searches (27.5%). One false negative was due to an incorrect procedure date (2.5%).

### Facility-level variation

**Fig. 1:**
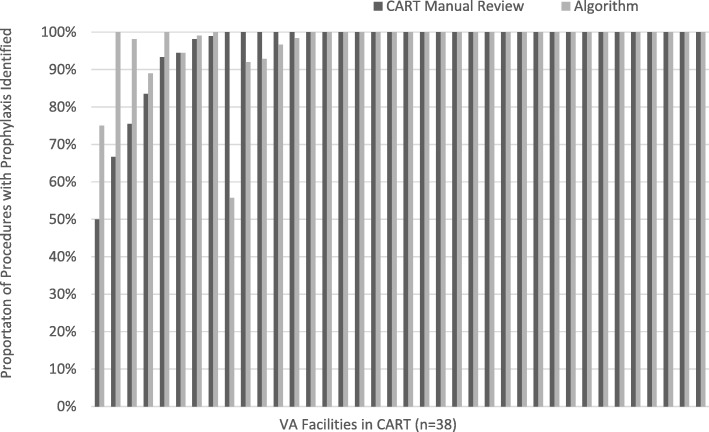
Algorithm Development: Facility Variation in Detection of Pre-Incisional Prophylaxis Among CART-EP Cardiac Device Procedures (*n* = 2102 procedures in 38 facilities)

**Fig. 2:**
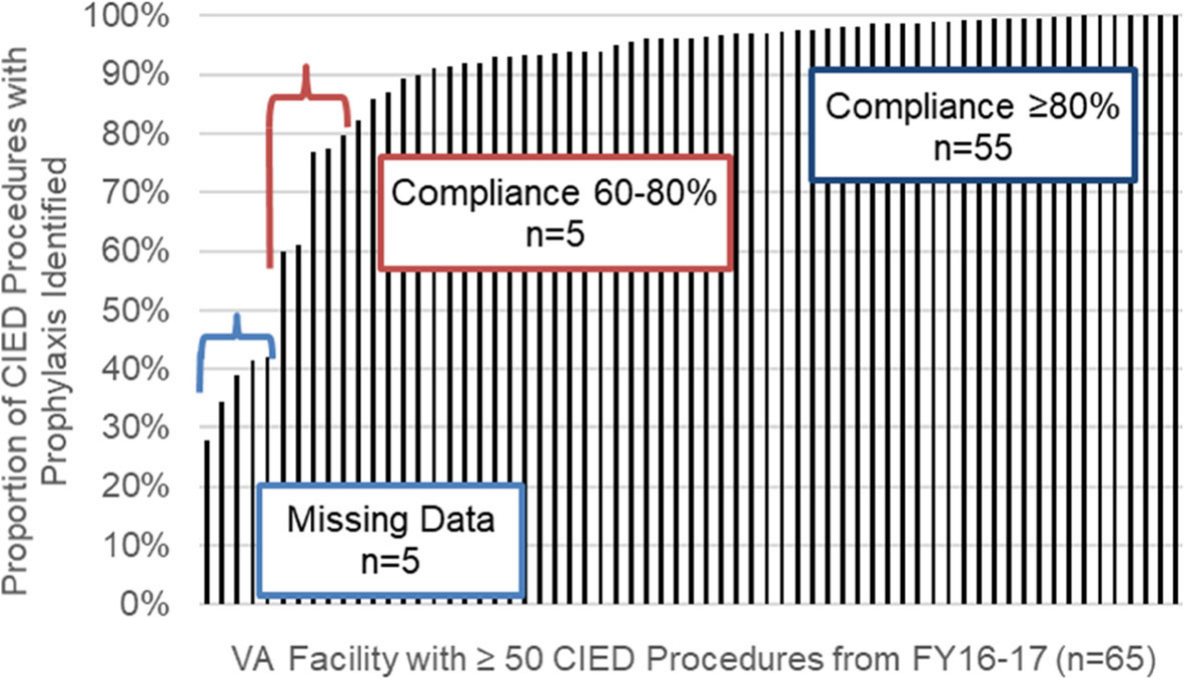
Algorithm Validation: Facility Variation in Detection of Pre-Incisional Prophylaxis Among Patients Undergoing Cardiac Device Procedures (*n* = 18,903 procedures in 65 facilities)

We found facility variation in both the development (Fig. 1) and validation data (Fig. 2). In comparison with CART-EP findings, in 25 of 38 facilities (66%), the algorithm and manual review results were identical, and compliance was 100%. In 8 facilities, the algorithm identified higher levels of compliance than the levels identified in the CART-EP review, whereas in 5 facilities the algorithm found lower compliance than the CART-EP manual review. In the validation data set, there was substantial variation on the facility level. Among 65 distinct VA facilities, 55 representing 15,450 (81.7%) of cases had prophylaxis identified in the algorithm in > 80% of cardiac device cases (Fig. 1); 5 facilities had prophylaxis compliance rates between 60 and 80% suggesting the algorithm may have accurately identified poor adherence to clinical guidelines, and 5 facilities had prophylaxis identified in < 60% of cases, suggesting that the algorithm may have low performance potentially due to clinical documentation practices that may limit the ability of an automated system to accurately measure pre-incisional prophylaxis, potentially due to ongoing reliance on hand-written notes in these facilities.

## Discussion

Appropriate pre-incisional antimicrobial prophylaxis is a cornerstone of prevention of procedure-related infections, including cardiac device infections. Prior work demonstrates that pre-incisional antimicrobial prophylaxis can reduce the incidence of infections by up to 80% [3, 18]. However, resources available for monitoring this key quality metric have been limited. To address this need, we created a novel clinical informatics tool to measure appropriate, guideline-concordant pre-incision antimicrobial prophylaxis for cardiac device procedures. Methodology developed can be adapted for application in other procedural settings, including for traditional surgical procedures to automate and expand quality monitoring activities. We found our electronic identification algorithm was highly accurate in most facilities; however, where EMR documentation included scanned hand-written notes, the algorithm found compliance rates of 60% or less and thus might not accurately measure quality in these settings. As EMR use increases in the VA, and hand-written notes are increasingly not used for clinical documentation, implementation of this tool could be used to expand quality measurement and reduce the burden of manual review to identify appropriate care, particularly in light of the transition to a Cerner EMR [19, 20].

We identified several potential barriers to disseminating this automated, clinical informatics tool throughout the VA – these concerns are likely to also affect non-VA facilities with modern EMRs. First, there was wide variety in how pre-incisional antimicrobial prophylaxis was documented. In one facility, no orders were placed and no specific antimicrobial was documented in the EMR; however, there was text in the clinical note that prophylaxis not otherwise specified had been administered. Thus, methods aimed to measure specific antimicrobial type will not be effective for capturing whether appropriate prophylaxis was given in these procedures. A potential way to adjust the algorithm is to include “prophylaxis” as a text search, however, this would also collect “no prophylaxis given” and “post-incision prophylaxis” as false-positives. Due to the potential for high rates of false positives with this term, and the limited value it demonstrated for improving algorithm performance, we elected not to use it in our tool.

Another reason for false-negative results was documentation only in scanned-in, hand-written paper records that did not include electronic text search functionality. Hand-written paper records may have been more common during the time-period of the development cohort (FY2005–15); thus, as EMRs and electronic anesthesia documentation is increasingly adopted, this may be less of an implementation challenge in the future as there are emerging technologies for converting text in scanned files and hand-written documentation is increasingly de-implemented [21]. Two cases were missed because the clinician procedure note was entered after the procedure date. Due to the high rate of post-procedural antimicrobials in this population, approaching 50% of cases [22, 23], we elected not to extend the timeframe of the text note searches to include the period after the procedure, as doing so would inappropriately flag guideline discordant post-procedural administration as guideline-concordant and would greatly increase the number of false positive cases.

The last substantial reason for false-negatives was an order placed far in advance of the procedure, potentially because the procedure was rescheduled in the interim. Increasing the timeframe for the searches to a period greater than 7 days prior to the intervention greatly increases the false positive rate, as the probability of an intervening antimicrobial prescription for another reason, unrelated to prophylaxis, greatly increases. Thus, altering the tool to capture these additional prophylaxis orders was not pursued. However, if the goal is to maximize sensitivity, then this would be a potential strategy to enhance case ascertainment.

False positive flags were attributed to several different factors. As noted above, inappropriate and ineffective post-procedural antimicrobial use is common in the cardiac device population [22, 24]. These post-incisional antimicrobials were often documented in the clinician note about the procedure and thus were identified during text searches of specific antimicrobial types. Limiting the false-positives that arise from post-incisional antimicrobials is challenging. The clinician note about the procedure is the place where prophylaxis is most commonly documented in the EMR and excluding it would create many more false-negative cases. Prior to adjustment of the antimicrobial list, several cases had a false-positive flag due to documentation of an antimicrobial allergy, typically a penicillin allergy, which is the most commonly documented antimicrobial allergy [25], in the clinician note. However, these antimicrobials are rarely used for prophylaxis, and after we removed them from the EMR text note searches, we found fewer false positives arising from allergy documentation.

The algorithm could be improved with better adherence to documenting antimicrobial orders and administration in existing structured data fields. The opportunity to create structured procedure or prophylaxis notes within the VA EMR may arise as the VA transitions to the Cerner EMR. Another way to improve algorithm performance may be through deep machine learning approaches. These methods create algorithms using a corpus of previously reviewed cases to train the computer to detect patterns of interest, and have been useful in isolating information like templated questions and answers documented in VA clinical notes [26]. .Application of these complex algorithms many not be practical given the facility variation in EMR documentation we discovered and the upcoming VA EMR transition [27]. This is a promising opportunity for future research.

Our study was limited in several ways. First, our initial algorithm was developed using the data from the VA CART-EP program, which had voluntary participation and only a subset of VA facilities and procedures were entered into the database. We attempted to address this limitation by validating the algorithm using a national dataset including all facilities and all cardiac device procedures. When the algorithm was applied to all VA facilities, we found substantial interfacility variation, which may cause implementation challenges if EMR documentation practices do not evolve. Applications developed for use within the VA EMR may not be directly applicable to other EMR systems; however, the programming used in our algorithm is relatively straightforward, and the most commonly used systems outside of the VA, including Cerner and EPIC, have text note searching options [19, 28]. Structured variables, such as antimicrobial orders, are generally easily extracted from any electronic order entry system [10].

## Conclusions

We developed a methodology based on structured and unstructured data elements from the EMR that could be applied for real-time quality measurement within the VA healthcare system. This method could be adapted for other procedural areas where antimicrobial prophylaxis is recommended but comprehensive measurement has been limited to resource-intense manual review.

### Supplementary information

**Additional file 1.** Description of Antimicrobial Search Terms.

## Data Availability

The data that support the findings of this study are not publicly available due to federal data policies; however, the authors can provide limited de-identified data upon reasonable request and with permission of the VA Boston Healthcare System Data Security and Privacy office.

## Appendix

**Table 3:** Algorithm components for measurement of pre-procedural antimicrobial use in cardiac device procedures

Electronic Medical Record Extract	Specific Fields	Criteria
Inpatient Utilization	○ CPT code	○ Matches CPT code for cardiac device procedure
	○ Procedure date+time	
	○ Inpatient procedure ID	
	○ Patient ID	
Outpatient Utilization	○ CPT code	○ Matches CPT code for cardiac device procedure
	○ Procedure date+time	
	○ Outpatient procedure ID	
	○ Patient ID	
Text Integrated Clinical Notes	○ Note entry date	○ Note date created ≥7 days of procedure ○ Note text matches wild card text string for antibiotic name (list of search terms in Additional file 1)
	○ Title	
	○ Document definition	
	○ Text	
	○ Patient ID	
Inpatient Pharmacy Dispensing	○ Drug dispensed date	○ Drug dispensed date ≤7 days of procedure date ○ Drug name matches wild card text string for antibiotic name (list of search terms in Additional file 1) ○ Days supply = 1
	○ Drug class code	
	○ Drug name without dose	
	○ Days supply	
	○ Patient ID	
Outpatient Pharmacy Dispensing	○ Drug dispensed date○	○ Drug dispensed date ≤7 days of procedure date ○ Drug name matches wild card text string for antibiotic name (list of search terms in Additional file 1) ○ Days supply = 1
	○ Drug class code	
	○ Drug name without dose	
	○ Days supply	
	○ Patient ID	
CPRS Orders	○ Entered date+time	○ Order start date+time ≤ 7 days of procedure date+time ○ Order item name matches text string for antibiotic name (list of search terms in Additional file 1)
	○ Order start date+time	
	○ Order stop date+time	
	○ Order item name	
	○ Patient ID	

We pulled data from electronic medical record extracts according to the following criteria. Note, VA specific variables to ensure the fields are populated with true data, as opposed to place holder text, are not shown as these are not useful outside the VA. Full code available from authors

## Abbreviations

Abx: Antimicrobial
CART-EP: VA Clinical Assessment Reporting and Tracking-Electrophysiology
CDW: Corporate Data Warehouse
EMR: Electronic medical record
NPV: negative predictive value
PPV: positive predictive value
VA: Veterans Health Administration
