# Supplementary material for: Novel methodology to measure pre-procedure antimicrobial prophylaxis: integrating text searches with structured data from the Veterans Health Administration’s electronic medical record
Source: BMC Med Inform Decis Mak. 2020 Jan 30;20:15. doi: 10.1186/s12911-020-1031-5 (PMC6993312; doi:10.1186/s12911-020-1031-5)
Supplement: Supplementary file 1 — Additional file 1. Description of Antimicrobial Search Terms. [file 12911_2020_1031_MOESM1_ESM.docx]

**Appendix 1. Description of Antimicrobial Search Terms**

1. List of antibiotics extracted from pharmacy databases (order and administration).

| NAFCILLIN |
| --- |
| PIPERACILLIN/TAZOBACTAM |
| CEFAZOLIN |
| CEFAZOLIN/DEXTROSE |
| CEFTRIAXONE |
| CEFTRIAXONE/DEXTROSE |
| CEFEPIME |
| CILASTATIN/IMIPENEM |
| ERTAPENEM |
| MEROPENEM |
| CLINDAMYCIN |
| CIPROFLOXACIN |
| CIPROFLOXACIN/DEXTROSE |
| LEVOFLOXACIN |
| DAPTOMYCIN |
| LINEZOLID |
| VANCOMYCIN |

1. List of antibiotics commonly used for prophylaxis (text strings applied to clinician notes about the procedure):

("cefazolin") or

                              ("vancomycin") or

                              ("clindamycin") or

                              ("clindamycin") or

                              ("linezolid") or

                              ("ceftriaxone") or

                              ("nafcillin") or

                              ("daptomycin") or

                              ("ancef") or

                              ("Cefacidal") or

                              ("Cefamezin") or

                              ("Cefrina") or

                              ("Elzogram") or

                              ("Faxilen") or

                              ("Gramaxin") or

                              ("Kefzol") or

                              ("Kefol") or

                              ("Kefzolan") or

                              ("Kezolin") or

                              ("Novaporin") or

                              ("Reflin") or

                              ("Zinol") or

                              ("Zolicef") or

                              ("vanco") or

                              ("clinda") or

                              ("cleocin") or

                              ("vancocin")

                              ';

1. Full list of antibiotics for clinician note text searches:

                              ("cefazolin") or

                              ("vancomycin") or

                              ("cefepime") or

                              ("piptazo") or

                              ("clindamycin") or

                              ("kanamicin") or

                              ("dicloxicillin") or

                              ("diclox") or

                              ("ertapenem") or

                              ("imipenem") or

                              ("mero") or

                              ("clindamycin") or

                              ("linezolid") or

                             ("ceftriaxone") or

                              ("nafcillin") or

                              ("daptomycin") or

                              ("ciprofloxacin") or

                              ("levofloxacin") or

                              ("doxycycline") or

                              ("cefuroxime") or

                              ("erta") or

                              ("imi") or

                              ("mero") or

                              ("meropenem") or

                              ("imipenem") or

                              ("ertapenem") or

                              ("pip/tazo") or

                              ("tazobactam") or

                              ("ancef") or

                              ("Cefacidal") or

                              ("Cefamezin") or

                              ("Cefrina") or

                              ("Elzogram") or

                              ("Faxilen") or

                              ("Gramaxin") or

                              ("Kefzol") or

                              ("Kefol") or

                              ("Kefzolan") or

                              ("Kezolin") or

                              ("Novaporin") or

                              ("Reflin") or

                              ("Zinol") or

                              ("Zolicef") or

                              ("vanco") or

                              ("clinda") or

                              ("cleocin") or

                              ("pipericillin") or

                              ("zosyn") or

                              ("vancocin")

                              ';
